# Multimorbidity, health and aging in Canada and Australia: a tale of two countries

**DOI:** 10.1186/s12877-016-0341-z

**Published:** 2016-09-23

**Authors:** Andrew Wister, Hal Kendig, Barbara Mitchell, Ian Fyffe, Vanessa Loh

**Affiliations:** 1 Gerontology Research Centre and Department of Gerontology, Simon Fraser University, 2800-515 Hastings Street, Vancouver, BC V6B 5K3 Canada; 2 Centre for Research in Ageing, Health, and Wellbeing and ARC CEPAR, Australian National University, Acton, ACT 2601 Australia; 3 Departments of Gerontology and Sociology/Anthropology, Simon Fraser University, 2800-515 Hastings Street, Vancouver, BC V6B 5K3 Canada; 4 School of Psychology, Brennan MacCallum Building (A18), University of Sydney, Sydney, NSW 2006 Australia

**Keywords:** Multimorbidity, Aging, Health outcomes, Cross-national

## Abstract

**Background:**

Multimorbidity has been recognized as a major public health issue, negatively affecting health-related quality of life, including physical, functional, mental, emotional, and social domains, as well as increasing health care utilization. This exploratory study examines selected health outcomes associated with multimorbidity across older age groups/cohorts and gender, comparing Canada and Australia.

**Methods:**

Data were drawn from the 2008/09 Canadian Community Health Survey and the 2009 Australian HILDA survey. Seven major chronic conditions were identical across the two data sets, and were combined into an additive measure of multimorbidity. OLS and logistic regression models were performed within age group (45-54, 55-64, 65-74, 75+) and gender to estimate associations between multimorbidity and several health-related outcomes, including: loneliness, life satisfaction, perceived health, mobility restriction, and hospital stays, adjusting for marital status, education and foreign born status.

**Results:**

Overall, country-level differences were identified for perceptions of loneliness, life satisfaction, and perceived health. Australians tended to experience a greater risk of loneliness and lower self-rated health in the face of multimorbidity than Canadians, especially among older men. Canadians tended to experience lower life satisfaction associated with multimorbidity than Australians. No country-level differences were identified for associations between multimorbidity and hospital stays or mobility limitations.

**Conclusions:**

The associations between multimorbidity and health are similar between the two countries but are variable depending on population, age group/cohort, and gender. The strongest country-level associations are for indicators of health-related quality of life, rather than health care or mobility limitation outcomes.

## Background

The onset, severity, clinical pathways and population health outcomes of aging with multiple chronic illness – what has been termed multimorbidity – has received increasing attention in recent years. Most persons aged 65 and over have at least one chronic illness or condition, and a majority have more than one chronic condition, resulting in multimorbidity being identified as an urgent public and community health concern [1]. For instance, in Canadian and Australian population health surveys, it has been estimated that between 50 % and 65 % of persons 65 and over report concurrent multiple chronic conditions, depending on the illnesses surveyed [2, 3]. While a majority of older adults rate their perceived health, well-being and daily functional ability (indicators of successful aging) as high even in the face of chronic conditions [4, 5], multimorbidity remains a major contributor to disability, deaths, and health care costs [6]. Cascading effects of multiple chronic health conditions [7] and their potentially synergetic deleterious health-related and social consequences [8, 9] make co-occurrence of illness a major public health issue, for instance, increasing functional disability, social isolation and health care utilization, as well as lowering psychological well-being. The health impacts of multimorbidity are further compounded by the combination of longer life expectancies, compression of morbidity, changing lifestyles, earlier and more intensive treatment, and for many countries, larger cohorts (i.e., baby boomers) transitioning into advanced ages [10, 11]. Thus, the examination of correlates and health outcomes of multimorbidity is important for understanding not only its population health burden, but also why some older adults appear to be more resilient than others [12]. This area of inquiry is particularly relevant given the age-old question of why some older adults appear to ‘live well,’ adapt, or age successfully in the face of stressful illness conditions such as disability [13–15]. While most research has examined objective health outcomes [1, 6], research findings are equivocal with respect to health-related quality of life (HRQOL), such as lower life satisfaction and increases in loneliness and social isolation with the accompanying feelings of stress, anxiety, loss of self-esteem, and alterations in social roles [4, 11].

One area in which a gap in knowledge exists pertains to cross-national comparisons and investigation of the prevalence and unique contexts in which multimorbidity occurs over the age span. Comparative national analyses of multimorbidity will also help to identify factors that facilitate illness coping and resilience, often embedded in the social determinants of health [16, 17], as well as the impact of multimorbidity on quality of life, other health outcomes and global disease burden. The present exploratory study aims to: 1) examine the associations between multimorbidity and HRQOL, functional disability and health care utilization across older age/gender cohorts; and 2) conduct cross-national comparative analyses across Canada and Australia. These countries were selected because of the availability of comparable population health data, as well as similar population health and aging characteristics and health systems.

### Literature review

#### How should we measure multimorbidity?

A seminal work [18], distinguished comorbidity and multimorbidity, noting that the former focuses on an index disease or condition, such as studying all persons with diagnosed hypertension, but also examining the presence of other concurrent illnesses. Multimorbidity, on the other hand, has been defined as the presence of two or more concurrent illnesses without choosing one as the index disease [11]. Systematic reviews of multimorbidity [19, 20] have found that prevalence measures of multimorbidity are found with increasing age but are variable depending on the measure used, especially clinical versus self-report measures. Given our interest in comparing self-reported population health survey data across diverse samples within two different national populations and their illness profiles, we focus on this subset of measures. Research has shown that self-report measures of multimorbidity demonstrate good validity and reliability in research [11, 21].

One approach that has been applied to the estimation of global disease burden has been the combination of more common and severe chronic conditions and to assess their influence on the prediction of health outcomes such as disability [10]. The authors discovered that particular disease constellations (especially first order combinations involving dementia, arthritis, diabetes and/or heart disease) revealed the strongest relationships with the Older Americans Resources and Services (OARS) functional status measure. Furthermore, they found significant age-gender variations in the correlations with functional status, suggesting the need to examine associations across these primary demographic factors. A second approach is to identify groups of comorbid conditions applying a two-way clustering framework using pairwise concordance statistics [22, 23]. In comparing the clustering method to others used in the literature, these authors have identified some degree of underestimation of comorbid disease effects on health-related outcomes. A third approach to measuring self-report multimorbidity data is to use an additive or weighted scale capturing all contemporaneous chronic conditions. A convergent construct validity study [11], compared six weighted and unweighted chronic condition measures as predictors of selected health status and utilization outcomes. The research showed that a simple additive count produces robust estimates of the association between multimorbidity and functional status, health care utilization, and health-related quality of life (HRQOL). A disease combination approach that does not weight the chronic conditions has the advantage of a more direct interpretation, but the disadvantage of potentially omitting the full impact of such conditions. However, this approach may underestimate multimorbidity associations with outcomes, albeit this limitation will be common across the populations. In this paper, we employ an additive scaling of chronic conditions that are reported in comparable indices across the two health surveys under investigation.

### Multimorbidity risk and health-related outcomes

Research shows that multimorbidity is a risk factor for a variety of adverse health-related outcomes. These include, for example, a truncated life span [24], limitations or loss of function [10, 25], episodic pain and lower perceived health [1], as well as increased health care utilization [11], such as hospital use, doctor visits, and medications. While a majority of older individuals experience multimorbidity, especially if they live into their 80s and beyond, most learn to cope with chronic conditions and maintain independence and quality of life^4^, although the combined effects of multiple chronic conditions can be debilitating for some older adults. Chronic conditions are often episodic in severity and symptomology, with often synergetic deleterious effects on pain, function, and HRQOL [14]. Recently, researchers have paid closer attention to the impact of multiple chronic conditions on HRQOL, such as lower life satisfaction, feelings of stress, anxiety, depression and loneliness, increased social isolation, loss of self-esteem, and alterations in social roles [1, 4, 6]. Thus, there are both objective and subjective dimensions of health that require examination as important health-related outcomes of experiencing multiple chronic conditions in middle and old ager age.

Yet, there is also evidence that, while many individuals receive treatment, adapt, and cope with the outcomes of multiple chronic conditions, others presenting the same conditions at the same period in life experience significant adversity and loss. These may be indicative of an underlying chronic condition resilience trait – the ability to recover from (or cope with) the adverse effects associated with combinations of chronic conditions [1, 11, 12]. Since there is research showing that not only illness resilience, but also symptomology, of chronic conditions is associated with age [26, 27], it is important to examine health indicators associated with multimorbidity across different age groups/cohorts. There are also variations in chronic illness or condition experiences and coping for older men and women, where women tend to experience more issues pertaining to altered appearances and negative effects on family relationships, whereas men tended to be more stoic [28].

### Cross-national multimorbidity contexts

Research into multimorbidity has been growing in recent years; however, there is virtually no research that examine multiple chronic conditions across different country contexts. While the prevalence of particular illnesses and their co-occurrence is likely to vary by country, region, and geography, the associations between multimorbidity and subjective and objective health outcomes may also vary. This is due to combinations of different health care systems and treatment protocols, health promotion and self-care programs and behaviours, and differences in other related healthy aging processes.

Recently, efforts have been made to rank countries in terms of key aspects of older people’s health and well-being. The 2015 Global AgeWatch Index [29] combines standard data on the following domains: 1) income security (pension coverage, poverty rate, relative welfare of older people, and GNI per capita); 2) health status (life expectancy at 60, healthy life expectancy at 60, and psychological well-being); 3) functional capability (employment of older people, and educational status); and 4) enabling environment to function independently (social connections, physical safety, civic freedom, and access to public transport). Of the 96 countries for which the index could be produced, Canada ranked 5^th^ whereas Australia ranked 17^th^ [29]. Ranking by domain showed Canada in the top 10 for all four domains: income security – 10th; health status – 4th; capability – 10th; and enabling society and environment – 9th. For Australia, the ranks were: income security – 62nd; health status – 5th; capability – 8th; and enabling society and environment – 26th. Based on this index, we would not expect a lot of difference between Canada and Australia in terms of multimorbidity or capability (work and education). However, Canada scores considerably higher in terms of income security and enabling society than Australia.

Since Canada and Australia share similar universal health care systems as well as health status indicators, we anticipate few differences in the prevalence of multimorbidity across the countries, in addition to the influence of multimorbidity specifically on health care utilization (e.g., hospital stays), after accounting for demographic factors. We also expect few differences in terms of associations between multimorbidity and mobility limitations, given that chronic illness or condition experiences are likely to be analogous in Canada and Australia, since the index capability scores are similar and that there are parallel health care treatment modalities and pathways. However, health-related quality of life indicators (e.g., perceived health, well-being, life satisfaction, and loneliness) may be experienced differently between the countries, possible related to income security and enabling environments being considerably better ranked in Canada than Australia. Income security and enabling environments have been connected to social isolation and loneliness, as well as life satisfaction [30, 31].

The current exploratory Canada-Australia comparative analysis examines the associations of multimorbidity and several health and health-related outcomes within four age groups/cohorts, and gender, and adjusting for selected demographic covariates.

## Methods

### The 2008/09 Canadian community health survey – healthy aging

Commissioned by Statistics Canada as part of the CCHS program, the CCHS – Healthy Aging is a unique cross-sectional dataset. The CCHS Healthy Aging collects information about the factors that influence healthy aging of Canadians aged 45 and over through a multidisciplinary approach^30^. Factors such as general health and well-being, chronic condition, perceived health, physical activity, use of health care services, social participation and other social determinants of health were collected. A total of 30,865 valid interviews were collected between December 2008 and November 2009, by way of a computer-assisted interviewing instrument. Approximately 94 % of interviews were conducted face-to-face by decentralized field interviewers who utilized the computer-assisted interviewing instrument [32]. Due to extenuating circumstances, the remaining interviews were collected over telephone. The target population were individuals aged 45 years or older living in both rural and urban areas. Excluded from the sample frame were individuals: living within the three less populated northern territories, living on First Nation reserves or Crown lands, living in institutions, living within some remote regions, or those who were employed full-time by the Canadian Forces. For the present study, we used the Public Use Sample File of the 2008/09 Canadian Community Health Survey available through the data liberation program of Canada. The original data was collected with full consent of all participants consistent with the Canada Statistics Act.

Population weights generated by Statistics Canada were utilized in order to account for sampling error by age, gender and geographic region. The weighted sample was rescaled to the original sub-sample size (*n* = 30,865) so that the analyses were not overpowered statistically.

### The 2009 Australian HILDA survey

This study uses data from Wave 9 (2009) of the Household, Income and Labour Dynamics in Australia (HILDA) Survey, which has been conducted annually since 2001. Initiated and funded by the Australian Government’s Department of Families, Housing, Community and Indigenous Affairs (now the Department of Social Services), HILDA is the only longitudinal study representative of Australian households. Although HILDA’s primary focus is on income dynamics, labour market dynamics, and family dynamics, it also collects information on general health and well-being factors such as chronic condition, perceived health, physical activity, social participation and other social determinants of health. Wave 1 (2001) began with a sample of 7682 households, comprising 19,914 individuals. All consenting individuals aged 15 years or older in the selected households are included in the yearly data collection [33].

Information is gathered via face-to-face interviews using a household form, a household questionnaire, and a person questionnaire, and a self-completion questionnaire. The household form includes basic information about household composition and is administered immediately after making contact with someone in the household. The household questionnaire, administered once per household, collects household information such as housing and childcare arrangements. The person questionnaire, administered to each consenting member of a household aged 15 years or older, collects personal information such as employment, income, family and background information. The self-completion questionnaire is left at each residence in order to be self-administered by consenting household members aged 15 years and older, and is either collected by the research interviewer at a later date, or returned by mail.

The HILDA study has been designed and managed by the Melbourne Institute of Applied Economic and Social Research at the University of Melbourne. Roy Morgan Research, a private market research company, has conducted the HILDA data collection since 2009. Previously, fieldwork for the project had been conducted by The Nielsen Company from 2001-2008.

A total of 9245 valid interviews were collected between August 2009 and March 2010 for Wave 9 (2009) of the HILDA survey. The sub-sample selected for this comparative study includes 5532 Australians aged 45 years or older, who completed all surveys, including the self-administered survey. Weights generated by the Melbourne Institute were utilized in order to account for sampling error. The weighted sample was rescaled to the original sub-sample size. Table 1 shows the CCHS and HILDA frequencies by age group (45-54, 55-64, 65-74, 75+), gender and country, weighted to each population and rescaled to the original sample sizes. While the distributions are similar across the countries, we observe statistically significant differences due to the large sample sizes.

**Table 1:** Frequencies by age group, gender and country, weighted and rescaled

Age	Women*		Men*		Total*	
	Canada (%)	Australia (%)	Canada (%)	Australia (%)	Canada (%)	Australia (%)
45 to 54	5963 (37.2)	1022 (35.5)	5915 (39.8)	953 (36.0)	11,878 (38.5)	1975 (35.7)
55 to 64	4632 (28.9)	866 (30.0)	4468 (30.1)	835 (31.5)	9100 (29.5)	1701 (30.7)
65 to 74	2845 (17.8)	558 (19.4)	2604 (17.5)	515 (19.4)	5449 (17.7)	1072 (19.4)
75 +	2581 (16.1)	437 (15.1)	1856 (12.5)	348 (13.1)	4437 (14.4)	784 (14.2)
Total	16,021	2882	14,844	2650	30,865	5532

*Note*. * *p* < .05 determined by Chi-square tests of different age distributions between countries

### Measurement

All of the variables used in this study were measured comparably for Canada and Australia, except for loneliness (shown below). Frequencies and descriptive statistics are shown for all variables in Table 2, and are similar across the samples, except where noted below.

**Table 2:** Frequencies for Canadian CCHS Survey (*n* = 30,865) and Australian HILDA survey (*n* = 5532), weighted and rescaled to sample size

Interval scales	Range		Mean		Standard deviation	
	Canada	Australia	Canada	Australia	Canada	Australia
Multimorbidity additive scale^a^	0 to 7	0 to 7	1.03	1.10*	1.12	1.19
Loneliness	3 to 9	1 to 7	3.77	2.55*	1.33	1.78
Life Satisfaction	0 to 10	0 to 10	7.97	7.95	1.72	1.49
Dichotomous variables	Categories		Frequency (%)			
			Canada (*n* = 30,865)		Australia (*n* = 5532)	
Gender	Female		16,021 (51.9)		2882 (52.1)	
	Male		14,844 (48.1)		2650 (47.9)	
Marital status	Single/Widowed/Divorced/Sep.		8109 (26.3)		1481 (26.8)	
	Married/Common-law		22,756 (73.7)		4051 (73.2)	
Foreign born status*	Foreign born - Canada/Australia		7734 (25.1)		1683 (30.4)	
	Native born - Canada/Australia		23,131 (74.9)		3850 (69.6)	
Education level*	Secondary or less		12,909 (41.8)		2566 (46.4)	
	More than secondary		17,956 (58.2)		2966 (53.6)	
Self-rated health*	Poor/Fair		4907 (15.9)		1293 (23.4)	
	Excellent/Very good/Good		25,958 (84.1)		4239 (76.6)	
Hospital stays in last year*	No		28,166 (91.3)		4720 (85.3)	
	Yes		2699 (8.7)		812 (14.7)	
Mobility limitation*	No		29,204 (94.6)		5314 (96.1)	
	Yes		1661 (5.4)		219 (3.9)	

*Notes*. ^a^The multimorbidity additive scale for Australia includes (*n* = 4832) valid cases due to missing

* *p* < .05 determined by Chi-square or *t*-tests of differences between countries

#### Independent variable

##### Multimorbidity

This primary independent variable was measured using self-reported diagnoses (yes/no) for the following seven chronic conditions that were collected in a closely comparable manner across the two surveys: arthritis/osteoporosis, asthma, blood pressure (hypertension), bronchitis/emphysema, cancer, diabetes, and heart disease. These were the only chronic conditions available in the HILDA data set except for stroke, which could not be used because it was measured differently across the samples. The small percentage of missing in both surveys were recoded to the mode, and we compared this method to omission of missing cases, which resulted in no differences to the findings. A 7 point additive scale was generated (range 0-7), based on positive responses to the above illness diagnoses. The mean number of chronic conditions was 1.03 (*SD* = 1.12) for Canada and 1.1 (*SD* = 1.19) for Australia, resulting a statistically significant difference. These prevalence rates are for persons aged 45 and over, are not based on all chronic conditions, and are therefore slightly lower than for other studies using only persons aged 65 and over ^2^. The use of self-reported illness has been shown to be reliable and valid ^11^. The seven chronic conditions included in this additive multimorbidity scale represent the most common chronic conditions affecting older adults, and are associated with limited mobility and function.

Table 3 shows the multimorbidity prevalence by age group, gender and country. The average number of these seven condition are similar across the countries, ranging from less than one condition for those aged 45-54 to two conditions for those 75+, demonstrating a high level of consistency. The only statistically significant differences are for women aged 75+ and men aged 55-64 across the countries, where the Australians report modestly higher rates.

**Table 3:** Mean and standard deviation of multimorbidity additive scale by age, gender and country*

Age	Women		Men	
	Canada	Australia	Canada	Australia
	Mean (Standard Deviation)	Mean (Standard Deviation)	Mean (Standard Deviation)	Mean (Standard Deviation)
45 to 54	.61 (.94)	.64 (.93)	.53 (.80)	.54 (.88)
55 to 64	1.08 (1.08)	1.13 (1.10)	.91 (1.02)	1.10 (1.12)*
65 to 74	1.55 (1.14)	1.64 (1.29)	1.39 (1.16)	1.35 (1.17)
75 +	1.85 (1.16)	2.03 (1.28)*	1.65 (1.22)	1.76 (1.32)

*Note.* * *p* < .05 determined by Chi-square tests of different age distributions between countries

#### Dependent variables

##### Loneliness

For the CCHS, we used the Hughes et al. [34] 3-item loneliness scale. This measure has been developed specifically for large scale health and social surveys of older adults and has been shown to have good psychometric properties. Responses included hardly ever, some of the time, and often for the following question items: 1) “How often do you feel that you lack companionship?”; 2) “How often do you feel left out?”; and 3) ”How often do you feel isolated from others?”. This resulted in a 7 point scale. The 2.9 % missing were recoded to the mean. For the Australian HILDA survey, loneliness was also measured using a 7-point response set (1 = strongly disagree, 7 = strongly agree) to the statement,” I often feel very lonely.” Comparisons among these and related loneliness scales in the literature indicate that the scales are highly correlated in both surveys [30–34]. A statistically significant difference was observed, where a higher loneliness score was reported in the Canadian sample (see Table 2).

##### Life satisfaction

This variable was measured using a single item 10 point response (0 = totally dissatisfied, 10 = totally satisfied) to very similar questions across the surveys. For the CCHS, respondents were asked the question, “Overall, how satisfied are you with your life?” For the HILDA, they were asked, “All things considered, how satisfied are you with your life?”

##### Self-rated health

This variable was measured using responses to the question: “In general, would you say your health is (poor, fair, good, very good, excellent)?” For the multivariate analyses, we dichotomized this outcome variable into poor/fair (0) and good, very good, excellent (1).

##### Hospital stay in last year

For both surveys, we dichotomized this variable into no hospital stays (0), and had one or more stays (1). This categorization was necessary due to different response sets of multiple stays across the surveys.

##### Mobility limitation

For the CCHS, this variable was dichotomized into those who do not need any assistive devices or require help with mobility (0) and those who reported needing help due to a disability or mobility limitation (1). For the HILDA survey, we used a similar split of the variable, where those who report no impairment (0), and those who report that they had a health condition, impairment or disability that restricts everyday activities and requires help with mobility (1).

#### Covariates

##### Marital status

This variable was dichotomized into widowed, separated, divorced, single (0), and married, common law (1). *Foreign born status* was divided into foreign born (0), and native born (1). Finally, *education level* was dichotomized into secondary or less (0), and more than secondary (1). Additional covariates were also examined, including income, body mass index, and home care utilization, but were removed from the analyses because they were found to not affect the primary associations between multimorbidity and the health-related outcomes.

### Analytic strategy

All of the analyses were conducted separately for four age groups (45-54, 55-64, 65-74, 75+) and gender (females, males). The within age group analyses mitigates the selection/survival effect problem associated with the study of health and age [4]. In addition, using these age groups allowed for investigation into potential age or cohort effects in the experiencing of multimorbidity on a variety of outcomes. Ordinary least squares (OLS) regression techniques were used to examine the relationships between multimorbidity and the two continuous dependent variables: loneliness and life satisfaction. These psycho-social outcomes have been considered as potentially influenced by the social determinants of health associated with both multimorbidity and the health outcomes under study [11, 35, 36]. Logistic regression analyses were used to examine multimorbidity associations with the dichotomous dependent variables: perceived health, hospital stays and mobility limitation. All multivariate analyses adjust for the three covariates: marital status, foreign born status, and education.

For the OLS regression, if the confidence intervals (CIs) for the unstandardized beta coefficients fell outside of the range for both countries, we determined that there were substantive differences (termed CI Method). This method accounts for different standard errors associated with each country. Since CIs are not available for standardized beta coefficients (which allow for relative comparison), we also report standardized beta coefficients. Standardized Z score conversion methods for determining differences in correlations for different samples [37, 38] were not used, since the large sample sizes result in statistically significant differences for coefficients with even very small variations across the countries. For the logistic regression analyses, if the CIs for the estimated adjusted odds ratios fell outside of the range for each country, we determined that there were substantive differences.

## Results

### Prevalence of major chronic conditions

Table 4 provides the prevalence rates for the seven chronic conditions separately by the four age groups (45-54, 55-64, 65-74, 75+) and gender for the two countries. The majority of illness prevalence rates are relatively similar for Canada and Australia; however, where differences occur, Canadians appear to report lower rates, except for heart disease and diabetes at the oldest ages. Only prevalence rates with greater than 5 % absolute point difference that are statistically significant are highlighted, since small percentage differences can result in statistically significant differences across the samples due to the large sizes (see Table 4). Among persons aged 45 to 54, the prevalence rates of asthma are significantly lower in Canada than Australia for both women (9.6 % and 15.6 %, respectively) and men (4.9 % and 10.1 %, respectively). Blood pressure rates are also slightly lower for women in Canada than in Australia (16 % and 22 %, respectively). None of the other chronic conditions reveal differences larger than 5 % (absolute point difference) between the two countries.

**Table 4:** Chronic illness prevalence by age group, gender, and country

Chronic condition	Women		Men		Women		Men	
	Canada (%)	Australia (%)	Canada (%)	Australia (%)	Canada (%)	Australia (%)	Canada (%)	Australia (%)
	45 to 54				55 to 64			
Arthritis & Osteoporosis	1130 (19.0 +/- .01)	211 (22.4 +/- .03)*	799 (13.5 +/- .01)	133 (14.5 +/- .02)	1986 (42.9 +/- .01)	355 (43.0 +/- .03)	979 (21.9 +/- .01)	289 (36.6 +/- .03)*
Asthma	571 (9.6 +/- .01)	146 (15.6 +/- .02)*	287 (4.9 +/- .01)	92 (10.1 +/- .02)*	425 (9.2 +/- .01)	129 (16.5 +/- .03)*	224 (5.0 +/- .01)	74 (9.8 +/- .02)*
Blood Pressure	955 (16.0 +/- .01)	209 (22.0 +/- .03)*	1058 (17.9 +/- .01)	173 (18.8 +/- .03)	1455 (31.4 +/- .01)	343 (41.8 +/- .03)*	1445 (32.3 +/- .01)	297 (37.5 +/- .03)*
Bronchitis & Emphysema	272 (4.6 +/- .01)	32 (3.5 +/- .01)	119 (2.0 +/- .00)	15 (1.7 +/- .01)	261 (5.6 +/- .01)	40 (5.2 +/- .02)	160 (3.6 +/- .01)	50 (6.7 +/- .02)*
Cancer	93 (1.6 +/- .00)	45 (4.9 +/- .01)*	102 (1.7 +/- .00)	31 (3.4 +/- .01)*	112 (2.4 +/- .00)	61 (7.9 +/- .02)*	117 (2.6 +/- .00)	81 (10.9 +/- .02)*
Diabetes	380 (6.4 +/- .01)	50 (5.4 +/- .01)	373 (6.3 +/- .01)	53 (5.8 +/- .02)	459 (9.9 +/- .01)	67 (8.6 +/- .02)	595 (13.3 +/- .01)	103 (13.6 +/- .02)
Heart Disease	240 (4.0 +/- .00)	36 (3.9 +/- .01)	368 (6.2 +/- .01)	41 (4.5 +/- .01)*	303 (6.5 +/- .01)	54 (6.9 +/- .02)	536 (12.0 +/- .01)	106 (13.8 +/- .02)
	65 to 74				75+			
Arthritis & Osteoporosis	1619 (56.9 +/- .02)	318 (61.3 +/- .04)	886 (34.0 +/- .02)	191 (38.5 +/- .04)	1695 (65.7 +/- .02)	284 (70.5 +/- .04)	770 (41.5 +/- .02)	179 (55.1 +/- .05)*
Asthma	261 (9.2 +/- .01)	102 (20.9 +/- .04)*	168 (6.5 +/- .01)	49 (10.4 +/- .03)*	231 (9.0 +/- .01)	63 (17.5 +/- .04)*	137 (7.4 +/- .01)	36 (11.8 +/- .04)*
Blood Pressure	1371 (48.2 +/- .02)	281 (54.0 +/- .04)*	1170 (44.9 +/- .02)	207 (42.2 +/- .04)	1522 (59.0 +/- .02)	260 (64.8 +/- .05)*	892 (48.1 +/- .02)	162 (50.8 +/- .05)
Bronchitis & Emphysema	202 (7.1 +/- .01)	37 (7.7 +/- .02)	146 (5.6 +/- .01)	34 (7.2 +/- .02)	198 (7.7 +/- .01)	19 (5.3 +/- .02)	131 (7.1 +/- .01)	31 (10.3 +/- .03)*
Cancer	127 (4.5 +/- .01)	57 (11.9 +/- .03)*	150 (5.8 +/- .01)	74 (15.4 +/- .03)*	110 (4.3 +/- .01)	65 (18.0 +/- .04)*	151 (8.1 +/- .01)	63 (20.5 +/- .05)*
Diabetes	438 (15.4 +/- .01)	60 (12.6 +/- .03)	552 (20.0 +/- .02)	94 (19.6 +/- .04)	377 (14.6 +/- .01)	64 (17.4 +/- .04)	366 (19.7 +/- .02)	51 (16.5 +/- .04)
Heart Disease	379 (13.3 +/- .01)	79 (16.3 +/- .03)	577 (22.2 +/- .02)	81 (16.9 +/- .03)*	649 (25.1 +/- .02)	112 (29.6 +/- .05)	624 (33.6 +/- .02)	99 (31.5 +/- .05)

*Notes.* The frequency of conditions in each age-sex-country subgroup is provided, as well as the percent and 95 % confidence interval in parentheses

*p < .05 determined by Chi-square tests of differences between countries within each age-sex subgroup

For those aged 55-64, arthritis/osteoporosis prevalence is considerably lower in Canada than Australia, but only for men (21.9 % and 36.6 %, respectively). Rates of asthma are again lower for Canadians at this age than for Australians for both women (9.2 % and 16.5 %, respectively) and men (5.0 % and 9.8 %, respectively). Also, blood pressure rates are lower in Canada than Australia for both women (31.4 % and 41.8 %, respectively) and men (32.5 % and 37.5 %, respectively) of this age group. Finally, reported rates of cancer are lower in Canada than Australia for both women (2.4 % and 7.9 %, respectively) and men (2.6 % and 10.9 %, respectively) of this age group.

Among those aged 65-74, rates of asthma are again lower for Canadians than for Australians, only among women (9.2 % and 20.9 %, respectively). In addition, blood pressure rates are lower in Canada than Australia, again among both women (48.2 % and 54.0 %, respectively). Cancer rates are also lower in Canada than Australia for both women (4.5 % and 11.9 %, respectively) and men (5.8 % and 15.4 %, respectively). This pattern reverses among men of this age group when comparing rates of heart disease, with Canadian men reporting more heart disease than Australian men aged 65-74 (22.2 % and 16.9 %, respectively).

For persons aged 75+, arthritis/osteoporosis prevalence is lower in Canada than Australia, but in this case only for men (41.5 % and 55.1 %, respectively). Rates of asthma also are lower for Canadians of this age than for Australians, again only among women (9.0 % and 17.5 %, respectively). Blood pressure rates for women in this age group are also modestly lower in Canada than in Australia (59.0 % and 64.8 %, respectively). Similar to findings at younger age groups, cancer rates are lower in Canada than Australia for both women (4.3 % and 18.0 %, respectively) and men (8.1 % and 20.5 %, respectively), likely related to the higher skin cancer rates in Australia.

### Multimorbidity and loneliness for Canada and Australia

Table 5 reports the unstandardized and standardized regression coefficients (betas) for multimorbidity and loneliness, by age group and gender. All analyses adjust for marital status, foreign born status and education. Also, all associations between multimorbidity and loneliness are statistically significant, except for Australian women aged 75 and over, where no association between multimorbidity and loneliness is observed.

**Table 5:** Unstandardized OLS regression coefficients for multimorbidity and loneliness by age, gender, and country

Age	Women				Men			
	Canada		Australia		Canada		Australia	
	B (β)	95 % CI	B (β)	95 % CI	B (β)	95 % CI	B (β)	95 % CI
45 to 54	.37*** (.26***)	.34, .41	.28*** (.16***)	.17, .40	.11*** (.07***)	.07, .15	.30*** (.16***)	.18, .42 ŧŧ
55 to 64	.13*** (.10***)	.09, .17	.09* (.06*)	-.02, .21	.10*** (.08***)	.07, .13	.31*** (.20***)	.20, .41 ŧŧ
65 to 74	.10*** (.08***)	.06, .14	.23** (.16**)	.10, .37	.08*** (.08***)	.05, .12	.09* (.07*)	-.03, .22
75 +	.13*** (.11***)	.08, .17	.02 (.01)	-.14, .17	.10*** (.10***)	.06, .14	.26** (.18**)	.10, .42

*Notes. CI* confidence interval, *B* unstandardized coefficient, *β* standardized coefficient, *ŧŧ* confidence intervals fall outside of country ranges

All coefficients were adjusted for marital-status, foreign born status, and education level. Higher coefficient indicates a greater association between the multimorbidity and loneliness

*p < .05. **p < .01. ***p < .001

As noted above, we use the CI Method of unstandardized beta coefficients to conclude country-level differences for the OLS results. We only report standardized betas for age-gender groups with country-level differences using the CI Method in order to all assessments of the relative size of the associations across countries. Comparisons of the confidence intervals (CI Method) of the unstandardized regression coefficients in Table 5 reveals only two instances in which the CIs do not overlap. Associations between multimorbidity and loneliness are considerably stronger for Australian men aged 45-54 and 55-64 (*β* = .16***, and *β* = .20***, respectively) than their Canadian counterparts (*β* = .07***, and *β* = .08***, respectively). In addition, while there is a weak statistically significant association between multimorbidity and loneliness for Canadian women aged 75+ (*β* = .11***), the relationship was not statistically significant for Australian women aged 75+. Overall, it appears that Australians (especially men) are at a greater risk of loneliness due to multimorbidity than Canadians.

### Multimorbidity and life satisfaction for Canada and Australia

The associations between multimorbidity and life satisfaction by age group and gender are presented in Table 6. There are four country level differences by age group and gender. The inverse associations between multimorbidity and life satisfaction are larger for Canadian women aged 55-64 and 65-74 (*β* = -.17***, and *β* = -.22***, respectively) than their Australian counterparts (*β* = -.08***, and *β* = -.13***, respectively). In addition, the negative associations between multimorbidity and life satisfaction are again stronger for Canadian men aged 65-74, as well as 75+ (*β* = -.23***, and *β* = -.21***, respectively) than for Australian men of those ages (*β* = -.08*, and *β* = -.12*, respectively). There is also a general pattern of considerably lower life satisfaction among the Canadian age-gender groups than their Australian counterparts. Thus, Canadians, especially older ones, appear to be at greater risk of experiencing lower life satisfaction in the face of multimorbidity than Australians.

**Table 6:** Unstandardized OLS regression coefficients for multimorbidity and life satisfaction by age, gender, and country

Age	Women				Men			
	Canada		Australia		Canada		Australia	
	B (β)	95 % CI	B (β)	95 % CI	B (β)	95 % CI	B (β)	95 % CI
45 to 54	-.35*** (-.20***)	-.40, -.31	-.28*** (-.19***)	-.38, -.19	-.30*** (-.15***)	-.35, -.25	-.38*** (-.22***)	-.49, -.27
55 to 64	-.27*** (-.17***)	-.31, -.22	-.11** (-.08*)	-.21, -.01ŧŧ	-.26*** (-.16***)	-.31, -.22	-.17*** (-.13***)	-.26, -.08
65 to 74	-.36*** (-.22***)	-.42, -.30	-.14** (-.13**)	-.24 -.04ŧŧ	-.34*** (-.23***)	-.39, -.28	-.10 (-.08)	-.22, .01ŧŧ
75 plus	-.21*** (-.13***)	-.27, -.15	-.19** (-.18**)	-.31, -.08	-.31*** (-.21***)	-.37, -.24	-.12* (-.12*)	-.24, -.01ŧŧ

*Notes. CI* confidence interval, *B* unstandardized coefficient, *β* standardized coefficient, *ŧŧ* confidence intervals fall outside of country ranges

All coefficients were adjusted for marital-status, foreign born status, and education level. Higher coefficient indicates a greater association between the multimorbidity and life satisfaction

*p < .05. **p < .01. ***p < .001

### Multimorbidity and self-rated health for Canada and Australia

The results of the logistic regression analyses between multimorbidity and the dichotomous self-rated health outcome (adjusting for marital status, foreign born status, and education level) are displayed in Table 7. All of the individual associations between multimorbidity and self-rated health are statistically significant (*p* < .001). The inverse associations (indicated by adjusted odds ratios that are below unity) between multimorbidity and self-rated health are similar across the countries, except for only one age/gender group. The likelihood of reporting good to excellent self-rated health (compared to poor/fair) is decreased for each increment of the multimorbidity scale by a larger factor (adjusted odds ratio = .57) for Canadian women aged 75 and over, compared to their Australian counterparts (adjusted odds ratio = .36). This indicates that perceived health is more affected by multimorbidity among Australian women 75 and over than Canadians of that age.

**Table 7:** Logistic regression adjusted odds ratios for multimorbidity and self-rated health by age, gender, and country

Age	Women				Men			
	Canada		Australia		Canada		Australia	
	AOR	95 % CI	AOR	95 % CI	AOR	95 % CI	AOR	95 % CI
45 to 54	.40***	.37, .44	.39***	.32, .48	.32***	.30, .36	.40***	.33, .48
55 to 64	.45***	.42, .49	.41***	.34, .50	.43***	.39, .46	.41***	.34, .49
65 to 74	.44***	.40, .48	.39***	.31, .49	.45***	.41, .49	.43***	.34, .53
75 plus	.57***	.53, .62	.36***	.28, .47 ŧŧ	.55***	.50, .60	.52***	.41, .65

*Notes. CI* confidence interval, *AOR* adjusted odds ratio, *ŧŧ* confidence intervals fall outside of country ranges

All coefficients were adjusted for marital-status, foreign born status, and education level. Higher coefficient indicates a greater association between the multimorbidity and perceived health

*p < .05. **p < .01. ***p < .001

### Multimorbidity and overnight hospital stays for Canada and Australia

Table 8 shows the results of the logistic regression analyses between multimorbidity and the dichotomous variable overnight stays at a hospital during the previous 12 months. All of the individual associations between multimorbidity and hospital stays are statistically significant (*p* < .001). lHowever, none of the CIs of the adjusted odds ratios fall outside of the range for each respective country, eading to a conclusion that there are no significant differences.

**Table 8:** Logistic regression adjusted odds ratios for multimorbidity and 12 month overnight hospital stays by age, gender, and country

Age	Women				Men			
	Canada		Australia		Canada		Australia	
	AOR	95 % CI	AOR	95 % CI	AOR	95 % CI	AOR	95 % CI
45 to 54	1.65***	1.52, 1.79	1.55***	1.29, 1.87	1.52***	1.36, 1.70	1.63***	1.30, 2.05
55 to 64	1.47***	1.34, 1.61	1.76***	1.46, 2.12	1.73***	1.58, 1.89	1.40***	1.16, 1.69
65 to 74	1.54***	1.40, 1.70	1.35**	1.12, 1.63	1.46***	1.32, 1.61	1.60***	1.31, 1.96
75 plus	1.44***	1.32, 1.58	1.31*	1.06, 1.61	1.41***	1.28, 1.56	1.52***	1.23, 1.88

*Notes. CI* confidence interval, *AOR* adjusted odds ratio, *ŧŧ* confidence intervals falls outside of country ranges

All coefficients were adjusted for marital-status, foreign born status, and education level. Higher coefficient indicates a greater association between the multimorbidity and overnight hospital stays within 12 months

*p < .05. **p < .01. ***p < .001

### Multimorbidity and mobility limitations for Canada and Australia

The results of the logistic regression analyses between multimorbidity and the dichotomous variable mobility limitations are presented in Table 9. Again, all of the individual associations between multimorbidity and hospital stays are statistically significant (*p* < .001). However, none of the CIs of the adjusted odds ratios fall outside of the range for each respective country, leading to a conclusion that there are no significant differences.

**Table 9:** Logistic regression adjusted odds ratios for multimorbidity and mobility limitation by age, gender, and country

Age	Women				Men			
	Canada		Australia		Canada		Australia	
	AOR	95 % CI	AOR	95 % CI	AOR	95 % CI	AOR	95 % CI
45 to 54	1.74***	1.54, 1.96	2.39***	1.89, 3.01	1.53***	1.25, 1.87	1.94***	1.46, 2.58
55 to 64	2.02***	1.79, 2.28	1.80***	1.45, 2.23	2.18***	1.88, 2.52	1.76***	1.44, 2.15
65 to 74	1.97***	1.74, 2.22	1.76***	1.41, 2.18	1.79***	1.55, 2.08	1.58**	1.21, 2.05
75 plus	1.52***	1.40, 1.65	1.84***	1.47, 2.30	1.44***	1.30, 1.59	1.91***	1.44, 2.53

*Notes. CI* confidence interval, *AOR* adjusted odds ratio, *ŧŧ* confidence interval is outside of country ranges

All coefficients were adjusted for marital-status, foreign born status, and education level. Higher coefficient indicates a greater association between the multimorbidity and mobility limitation

*p < .05. **p < .01. ***p < .001

## Discussion

A majority of older adults experience one or more concurrent chronic conditions, which often pose challenges for individuals due to their potentially cumulative and synergetic effects [1, 8, 9]. This exploratory paper examined associations between a standard additive scale measuring multimorbidity of seven major chronic conditions and selected subjective and objective health outcomes comparing Canadian and Australian middle-aged and older cohorts and by gender. The chronic conditions that were comparatively measured in the surveys included: arthritis/osteoporosis, asthma, blood pressure (hypertension), bronchitis/emphysema, cancer, diabetes, and heart disease. Within the four age groups (45-54, 55-64. 65-74. 75+) and gender, the majority of illness prevalence rates are relatively similar for Canada and Australia; however, where differences occur, Canadians appear to report lower rates, except for heart disease and diabetes at the oldest ages, where Australians report higher rates.

An additive multimorbidity scale was developed to examine the relationships between multimorbidity and five health outcomes (loneliness, life satisfaction, perceived health, hospital stays and mobility limitation) by age group and gender. The mean and standard deviation of the additive scale was similar across countries, although it does not account for variations in constellations of chronic conditions across populations. Several associations between multimorbidity and health outcomes were uncovered by age group and gender, and by country. Overall, the majority of country-level differences were identified for perceptions of loneliness and life satisfaction, and these were age and gender specific. The risk of experiencing loneliness associated with multimorbidity is higher for Australians than for Canadians, with country-level differences supported among men aged 45-54 and 55-64. This may be indicative of weaker support networks among Australian men living with multiple chronic conditions. Interestingly, the opposite effect is found when examining associations of multimorbidity on life satisfaction, where several Canadian age-gender groups were found to report considerably lower life satisfaction than Australian counterparts in the face of multimorbidity. These relationships are most prevalent among the older groups aged 55 and over for both men and women. This suggests that multimorbidity may be more detrimental to Canadians than Australians in terms of generalized well-being or associations possibly with resilience [12, 35] and is consistent with the rankings produced by the 2015 Global AgeWatch report [29]. However, while our data suggest a pattern of higher resilience to multimorbidity among Australians than Canadians, it appears to be highly age and gender specific. The associations between multimorbidity and self-rated health revealed only one country-level difference, where among persons aged 75+, Australian women reported considerably lower self-rated health than Canadian women of that age group. In addition, no country-level differences were identified for associations between multimorbidity and hospital stays or mobility limitations, as could be hypothesized based on the comparative ranking of the Global AgeWatch index domains along with other similarities in the population and health systems.

A number of limitations should be noted. First, the multimorbidity scale is an additive scale based on self reports of only seven chronic conditions, albeit major ones. Research is needed that examines different combinations of illnesses (such as recent statistical approaches to identify clusters of illnesses [22, 23], as well as clinical measures that capture the onset, length and severity of multimorbidity. Second, the data sets used for the comparative analyses between Canada and Australia were collected using different sampling and data collection strategies, are therefore prone to measurement error. While a conservative method to establish country-level differences were applied, further research is needed to substantiate the results. This should include adjusting for a larger set of variables that are measured consistently across the populations. Third, the loneliness dependent variable was measured differently across the populations; therefore, results should be interpreted with caution. Fourth, comparisons to other countries may shed further light on the health, health-care and social contexts that underlie the multimorbidity patterns found in this study. However, caution needs to be applied in extending comparisons beyond countries, like Canada and Australia, that are broadly at similar stages of socio-economic development with comparable cohort exposures of the age groups [39, 40]. For instance, a developing county may have less favourable socio-economic conditions and more disease exposure through the life span, different patterns of selective mortality, and fewer treatment options and support systems available to mitigate the association of multimorbidity on health-related quality of life; and cultural factors, for example in the meanings of self-rated health, can be of over-riding importance [40, 41]. Finally, a more comprehensive set of health and health-related outcomes of multimorbidity with improved measurement and adjustment for covariates (such as lifestyle factors, age, and income) would help to elucidate our findings. Future research needs to be conducted to investigate how multimorbidity reflects different exposures over the life span and differentially affects national populations and subgroups.

## Conclusion

Examination of chronic condition patterns in this study indicates primarily parallel trends in prevalence for Canada and Australia, except for higher rates of heart disease, diabetes and cancer among Australians. We also found several county-level differences in associations between multimorbidity and selected health-related outcomes, in particular, a higher risk of experiencing loneliness for older aged Australians (especially males) than for Canadians with multimorbidity, and alternatively, significantly lower reported life satisfaction among older Canadians than Australian counterparts in the face of multimorbidity. Overall, the findings indicate the national context of ageing and health is comparable between the countries, suggesting that findings from either country could have application to the other in epidemiological and health service research as well as health promotion and other interventions.

The exploratory findings on country differences suggest directions for further understanding social and environmental determinants of health and directions for responses in health promotion and services. For example, the higher rate of cancer in Australia, especially among men, may reflect the higher sun exposures and consequent melanomas, indicating the priority for limiting these exposures. The life satisfaction differences may indicate the potential for better understanding the impacts of climate and weather patterns in Canada as limiting factors in social support and community integration. The lower self-reported health of older Australian women with advancing age does not have any easy explanations and requires careful methodological and aetiological investigation as per previous cross-national comparisons between Australia and the United States^38.^ Other findings such as higher loneliness prevalence among older Australian men, and the age and gender specificity of the findings, suggest promising directions for better understanding the complex social and cultural context of chronic illness, its consequences, and avenues for prevention and amelioration.

## Abbreviations

CCHS: Canadian Community Health Survey
CI Method: Confidence interval method
HILDA: Household Income Labour and Dynamics Survey in Australia
HRQOL: Health-related quality of life.
